# The Role of *FTO* and Vitamin D for the Weight Loss Effect of Roux-en-Y Gastric Bypass Surgery in Obese Patients

**DOI:** 10.1007/s11695-015-1644-4

**Published:** 2015-02-28

**Authors:** Marcus Bandstein, Bernd Schultes, Barbara Ernst, Martin Thurnheer, Helgi B. Schiöth, Christian Benedict

**Affiliations:** 1 Department Neuroscience, Functional Pharmacology, Uppsala University, Box 593, 751 24 Uppsala, Sweden; 2 Interdisciplinary Obesity Center, eSwiss Medical & Surgical Center, St. Gallen, Switzerland

**Keywords:** Vitamin D, FTO, RYGB, Weight loss, Bariatric surgery

## Abstract

**Background:**

A recent study in children demonstrated that the rs9939609 single-nucleotide polymorphism in the fat mass and obesity (*FTO*) gene influences prospective weight gain, however, only in those who were vitamin D-deficient. If this might also be the case for Roux-en-Y gastric bypass (RYGB), surgery-induced weight loss is however unknown. The objective of this study is to examine if the magnitude of RYGB surgery-induced weight loss after 2 years depends on patients’ *FTO* rs9939609 genotype (i.e., TT, AT, and AA) and presurgery vitamin D status (<50 nmol/L equals deficiency).

**Methods:**

Before and at 24 months after RYGB surgery, BMI was measured in 210 obese patients (mean BMI 45 kg/m^2^, 72 % females). Serum 25-hydroxyvitamin D3 levels were also repeatedly measured. Following surgery, vitamin D was supplemented. Possible weight loss differences between genotypes were tested with multiple linear regressions.

**Results:**

The per-allele effect of each *FTO* A-allele on excessive BMI loss (EBMIL) was 3 % (*P* = 0.02). When split by baseline status, the EBMIL of vitamin D-deficient patients carrying AA exceeded that of vitamin D-deficient patients carrying TT by ~14 % (*P* = 0.03). No such genotypic differences were found in patients without presurgery vitamin D deficiency. Post-surgery serum levels of vitamin D did not differ between groups.

**Conclusions:**

Our data suggest that presurgery vitamin D levels influence the size of genotype effects of FTO rs9939609 on RYGB surgery-induced weight loss in obese patients.

## Introduction

Large cross-sectional studies have demonstrated that both children and adults carrying the rs9939609 obesity A-allele within an intron region of the fat- and obesity-associated gene (*FTO*) exhibit higher BMI scores and are more often obese than noncarriers [1–4]. In addition, a recent study involving 1,088 children demonstrated that carriers of the *FTO* A-allele gained more weight than noncarriers during a ~5-year observation period [5]. Complementing its important role for human body weight dynamics, some studies have demonstrated that *FTO* rs9939609 (or proxy single-nucleotide polymorphisms) could also partially account for interindividual differences in weight loss upon Roux-en-Y gastric bypass (RYGB) surgery [6–8]. Most interestingly, in the aforementioned study investigating the impact of *FTO* on weight gain during childhood, genotypic differences in weight gain were only seen between vitamin D-deficient children [5]. This suggests that the magnitude by which *FTO* influences human weight regulation may partially depend on nutritional factors, comprising vitamin D. However, as of yet, no study has investigated if the influence of *FTO* on RYGB surgery-induced weight loss is modulated by a patient’s presurgery vitamin D status. Thus, the present study aimed at investigating if the *FTO* A-allele predicts the 2-year weight loss response following RYGB surgery and if this depends on a patient’s vitamin D status. Importantly, following surgery, patients received vitamin D supplements to avoid vitamin D undersupply.

## Material and Methods

### Patients

RYGB surgeries and follow-up investigations were performed at the Interdisciplinary Obesity Center, St. Gallen, Switzerland. Before surgery, all patients (*n* = 210, 72 % females) were obese (BMI 45.2 ± 0.43 kg/m^2^), and none had undergone any kind of bariatric surgery procedure (e.g., gastric banding) before. Two different variants of RYGB surgery were performed, i.e., proximal and distal RYGBs [5, 9]. In both procedures, the largest part of the stomach was transected, and a small gastric pouch of about 20–30 mL was anastomized to the proximal jejunum with the diameter of the pouch–jejunal anastomosis standardized to be about 12 mm. In the proximal RYGB procedure, the biliopancreatic limb was side to side anatomized to the jejunum 150 cm distal from the pouch–jejunal anastomosis (Roux-en-Y limb length, 150 cm). In the distal RYGB procedure, the biliopancreatic limb was side to side anatomized to the ileum 60 to 100 cm proximal from Bauhin’s valve (common channel, 60–100 cm). The length to the biliopancreatic limb was approximately 60 cm in the proximal and 60 to 100 cm in the distal RYGB procedure.

### Assessments

All participants were genotyped for the *FTO*-associated single-nucleotide polymorphism (SNP) rs9939609 using DNA isolated from whole blood with a custom Illumina iSelect genotyping array (99.5 % success rate). Rs9939609 was confirmed to be in Hardy–Weinberg equilibrium.

At baseline (i.e., close to surgery), blood samples were drawn in the morning (0800–1100h) after an overnight fast, and serum 25-hydroxyvitamin D3 levels were determined by high-performance liquid chromatography (Chromsystems, Instruments & Chemicals GmbH, Munich, Germany). In general, serum levels of 25-hydroxyvitamin D3 lower than 50 nmol/L are defined as vitamin D deficiency [10]. Since 25-hydroxyvitamin D3 levels exhibit seasonal variation in obese humans [11], the date where the blood was collected was recorded. Post-surgery, all patients received standard oral vitamin D3 supplements (1,200 IU/day). If serum levels of 25-hydroxyvitamin D3 were below 50 nmol/L at follow-up investigations (i.e., at +3, +6, +9, +12, +18, and +24 months), patients received additional intramuscular injection of 300,000 IU every 3 months. Further preoperative preparations and post-operative follow-up procedures are described in more detail in [9].

At baseline and 24 months after RYGB surgery, height and weight were measured with patients wearing light clothing and no shoes. BMI was defined as weight (kg) divided by height squared (m^2^). BMI was utilized to calculate relative excessive BMI loss (EBMIL, cutoff for normal-weight BMI = 25 kg/m^2^) by the following equation [12]: 1 − ((initial BMI − final BMI)/(initial BMI − 25))) × 100.

### Statistics

A univariate general linear model (SPSS Statistics, version 21.0 for Windows, IBM, Chicago, IL, USA) was used to perform the regression analysis (assuming an additive model). Predictors of interest were the three-level *FTO* rs9939609 genotype (TT = 0, AT/TA = 1, AA = 2), baseline serum vitamin D levels, and their interaction term. In case of a significant interaction of *FTO* with baseline serum vitamin D levels, differences between genotypes were specified by post hoc ANCOVA analysis. Linear mixed effect models were used to investigate if *FTO* genotype groups, split by baseline vitamin D status, would exhibit post-surgery differences in the time course of serum levels of this micronutrient (i.e., comprising measurements at 3, 6, 12, 18, to 24 months). All analyses were adjusted for age, sex, and BMI at baseline, unless otherwise specified. Distal RYGB surgery is likely to cause stronger malabsorption of vitamin D than the proximal surgery procedure because the Y-connection is formed much closer to the lower end of the small intestine. Given that the vitamin D status was a variable of particular interest in our study, we also covaried for the surgery type in our analyses. The date where patients’ baseline session took place, comprising the measurement of circulating 25-hydroxyvitamin D3 levels, was utilized as nominal variable in our statistical models to account for seasonal variations of this vitamin (subdivided into six time intervals, i.e., January–February, March–April, May–June, etc.). Overall, a *P* value less than 0.05 was considered significant. Data are shown as mean ± SEM, unless otherwise specified.

## Results

As summarized in Table 1, approximately 70 % of our obese patients carried at least one copy of the *FTO* A-allele. In addition, ~50 % of our obese patients were vitamin D-deficient at baseline. Following RYGB surgery, patients exhibited, on average, an EBMIL of 83 %.

**Table 1:** Patients’ characteristics

Sex, *n* (% cohort)	
Female	151 (71.9)
Male	59 (28.1)
Age^a^ (years)	42.8 ± 0.8
BMI^a^ (kg/m^2^)	45.2 ± 0.4
Waist circumference^a^ (cm)	129 ± 2
Diabetes^a^, *n* (% cohort)	17 (8.1)
Serum triglyceride levels^a^ (mmol/L)	1.8 ± 0.1
Serum levels of vitamin D3^a^ (nmol/L)	52.9 ± 1.9
Vitamin D deficient, *n* (% cohort)	
Pre-surgery^a^	104 (49.5)
Post-surgery^b^	18 (9.1)
FTO rs9939609, *n* (% cohort)	
TT	57 (27.4)
AT	98 (46.7)
AA	55 (26.2)
Surgery type, *n* (% cohort)	
Distal RYGB	158 (75.2)
Proximal RYGB	52 (24.8)
BMI^b^ (kg/m^2^)	28.6 ± 3.9
EBMIL^b^ (%)	83.4 ± 1.1

If not otherwise described, data are mean±SEM. Blood to determine serum vitamin D levels and FTO rs9939609 genotype was collected in a fasted state. Serum levels of 25-hydroxyvitamin D3 lower than 50 nmol/L were defined as vitamin D deficiency. The presence of diabetes was defined as use of oral hypoglycemic agents or insulin

BMI body mass index, *EBMIL* excessive BMI loss, *SEM* standard error of mean, *RYGB* Roux-en-Y gastric bypass

^a^At baseline

^b^At +24 months post-Roux-en-Y gastric bypass surgery

### Association of FTO and Presurgery Vitamin D Status with 2-Year Weight Loss Induced by RYGB Surgery

**Fig. 1:**
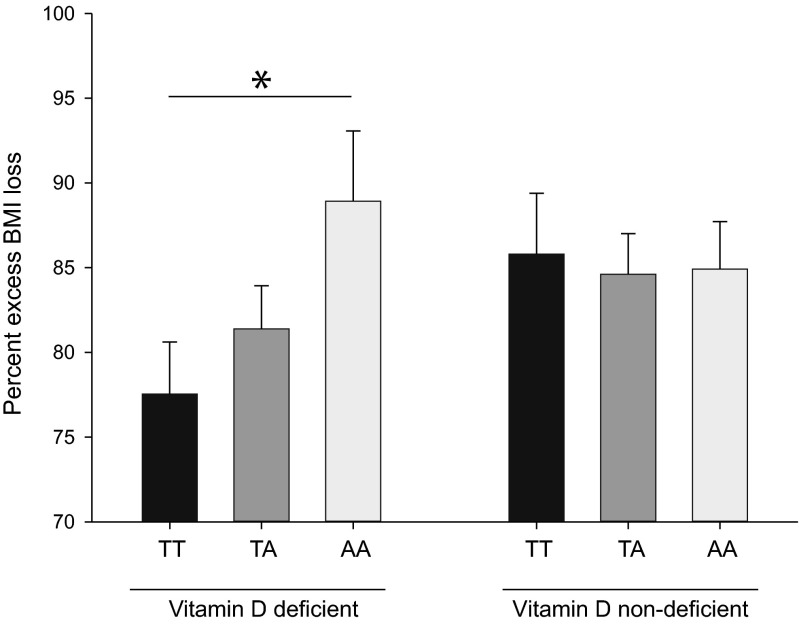
Percent excess BMI loss at 24 months post-surgery, split by patients’ FTO rs9939609 genotype and presurgery vitamin D status. A univariate general linear model was utilized to investigate if the FTO rs9939609 genotype (i.e., AA, AT, or TT) predicts the magnitude of 2-year weight loss following Roux-en-Y gastric bypass surgery and if this depends on a patient’s baseline vitamin D status (assuming an additive model). Baseline serum levels of 25-hydroxyvitamin D3 < 50 nmol/L were defined as vitamin D deficiency. Data are shown as mean±SEM. *P < 0.05 for pairwise comparisons between groups

A univariate general linear model analysis revealed a positive association between the 2-year EBMIL upon RYGB surgery and *FTO* rs9939609 genotype (independent variable coefficient *B*_(AA)_ = 20.7, *B*_(TA/AT)_ = 13.3, *B*_(TT)_ = 0 (reference value), *P* = 0.02). The per-allele effect of each *FTO* A-allele on excessive BMI loss (EBMIL) was 3 % (EBMIL, AA, 86.1 ± 2.3 %; TA/AT, 83.0 ± 1.7 %; TT, 81.5 ± 2.3 %; *P* = 0.02). The strength of this association, however, was influenced by presurgery serum levels of 25-hydroxyvitamin D3 (*P* = 0.04 for the interaction term “FTO*Vitamin D levels”). When split by their baseline vitamin D status, AA patients who were vitamin D-deficient exhibited a surgery-induced EBMIL that was ~14 % higher than that of vitamin D-deficient TT carriers (*P* = 0.03, Fig. 1, left panel). In contrast, no such genotypic differences were found in patients without presurgery vitamin D deficiency (*P* = 0.89), Fig. 1, right panel). Furthermore, no difference in EBMIL between vitamin D-deficient TT carriers and nondeficient TT carriers was seen (*P* = 0.23). Finally, the presurgery vitamin D status was not independently linked to EBMIL (*P* = 0.81).

### Serum Levels of Vitamin D Prior to and After Surgery

**Fig. 2:**
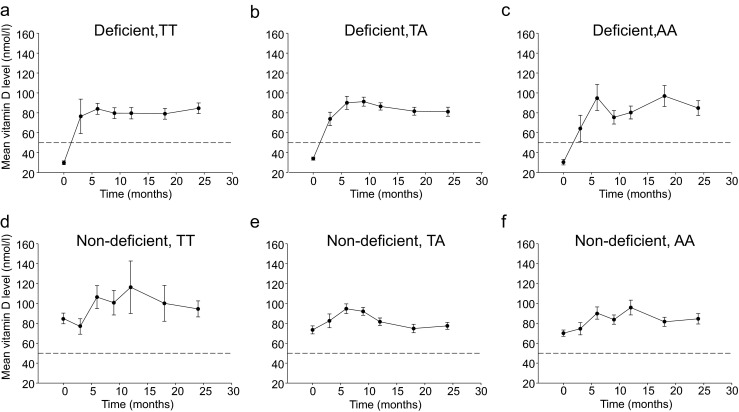
Serum levels of 25-hydroxyvitamin D3 in patients before and after RYGB surgery, split by FTO rs9939609 genotype and presurgery vitamin D status. Note that post-surgery, all patients received standard oral vitamin D3 supplements (1,200 IU/day). If serum levels of 25-hydroxyvitamin D3 were below 50 nmol/L (*dashed lines*) at follow-up investigations (i.e., at +3, +6, +9, +12, +18, and +24 months), patients received additional intramuscular injection of 300,000 IU every 3 months. Data are shown as mean±SEM

Baseline serum levels of 25-hydroxyvitamin D3 revealed that 49.5 % of the patients in our cohort were vitamin D-deficient, compared to 9.1 % who were vitamin D-deficient 2 years after surgery (*P* < 0.0001). A Fisher’s exact test showed that serum 25-hydroxyvitamin D3 levels after surgery did not differ between *FTO* genotype groups, when stratified by baseline vitamin D status (*P* > 0.05 at all time points post-surgery; Table 2). As seen in Fig. 2, the mean vitamin D level increased quickly in the vitamin D-deficient groups after surgery (and initiation of vitamin D supplementation). Among the presurgery deficient patients, 30 % remained deficient at the first follow-up visit (3 months post-op) and 11 % after 24 months. Some patients with baseline levels above the deficiency cutoff were deficient 24 months after surgery.

**Table 2:** Number of vitamin D-deficient patients before and at +3, +6, +9, +12, +18, and +24 months post-Roux-en-Y gastric bypass surgery, split by FTO rs9939609 genotype and presurgery vitamin D status

Months	*n* (*N*)			*n* (*N*)			*P* value
	*TT* ^a^	*TA* ^a^	*AA* ^a^	*TT*	*TA*	*AA*	
Baseline	35 (35)	50 (50)	19 (19)	0 (22)	0 (48)	0 (36)	–
+3	6 (17)	6 (24)	2 (12)	1 (8)	4 (25)	4 (15)	0.75
+6	1 (21)	2 (30)	1 (9)	1 (13)	1 (30)	1 (24)	0.88
+9	2 (19)	0 (32)	1 (10)	1 (12)	0 (29)	1 (22)	0.09
+12	2 (22)	2 (40)	1 (13)	1 (13)	1 (34)	1 (27)	0.84
+18	2 (23)	3 (40)	0 (14)	2 (15)	4 (39)	1 (25)	0.80
+24	5 (34)	4 (42)	1 (18)	2 (21)	3 (47)	3 (35)	0.87

Vitamin D deficiency was defined as serum levels of 25-hydroxyvitamin D3 < 50 nmol/L. Note that following Roux-en-Y gastric bypass surgery, all patients received standard oral vitamin D3 supplements (1,200 IU/day). If serum levels of 25-hydroxyvitamin D3 were below 50 nmol/L at follow-up visits, patients received additional intramuscular injection of 300,000 IU every 3 months. *P* values derive from Fisher’s exact test

*n* number of patients who were vitamin D-deficient, (*N*) number of patients for whom blood samples were available to determine their serum vitamin D status

^a^Vitamin D-deficient patients before surgery (i.e., at baseline)

## Discussion

Here, we show that patients who were vitamin D-deficient before surgery exhibited a ~14 % higher RYGB surgery-induced weight loss when they carried two copies of the A-allele in the fat mass and obesity (*FTO*) gene rs9939609, compared to vitamin D-deficient patients who were homozygous for the *FTO* T-allele. In contrast, no such differences were found between genotypes when presurgery serum vitamin D levels were above 50 nmol/L (i.e., their vitamin D status was considered sufficient). These results could suggest that presurgery vitamin D modulates the strength by which *FTO* predicts RYGB surgery-induced weight loss in obese humans. However, unless independent cohorts can replicate our findings, caution is needed before generalizing our results to patients undergoing other types of gastric bypass surgery (e.g., gastric banding).

Large cross-sectional genome-wide association studies have demonstrated that humans who carry the rs9939609 A-allele have higher BMI scores than noncarriers [1–4]. Each *FTO* risk allele increases BMI by 0.26–0.66 kg/m^2^, equivalent to ~0.84–2.1 kg in body weight for a 1.80-m-tall person, and the risk of obesity by 1.25–1.32 odds [13]. With these findings in mind, it might appear counterintuitive at first glance that our study demonstrates that obese patients who carried the rs9939609 A-allele exhibited the highest EBMIL after RYGB surgery. However, our findings receive also some support from the literature. For instance, in a study involving 520 obese patients, bariatric surgery-induced weight loss was greatest in those who were carrying the rs9939609 A-allele [6]. In addition, a previous nonsurgical study where obese patients participated in a hypocaloric diet demonstrated that carriers of the rs9939609 A-allele lost more body weight than noncarriers during the intervention period [14]. However, there are also studies that failed to show an impact of FTO on weight loss response to dietary interventions. For instance, in one study involving 204 Japanese women, *FTO* rs9939609 did not significantly change body weight or metabolic risk factors in response to calorie restriction [15].

The main finding of our study is that patients with a preoperative vitamin D deficiency lost more weight following RYGB surgery when they carried two copies of *FTO* rs9939609 A-allele, than their homozygote counterparts did, i.e., TT carriers who had a vitamin D deficiency. If replicated by others, these findings suggest that the effect of *FTO* on body weight regulation in humans is modulated by vitamin D. In line with our observation, a previous genetic study demonstrated that children who carried the *FTO* A-allele showed a greater weight gain over 5 years compared to noncarriers, however, only in those who were vitamin D-deficient [5]. This suggests that vitamin D may possess biological properties that can regulate the magnitude by which *FTO* or its genetic network [16, 17] impacts body weight regulation in humans. Vitamin D fulfills many biological functions in humans, comprising whole-body calcium metabolism [18]. A recent study has shown that *FTO* interacts with three isoforms of calcium-dependent protein kinase II: α, β, and γ. These protein kinases phosphorylate a broad range of substrates, including factors involved in the regulation of food intake and energy homeostasis (e.g., BDNF, NPY1R) [19]. With this in mind, it could be speculated that *FTO* A-allele carriers who were deficient for vitamin D before RYGB surgery (a condition that is expected to concur with a low calcium bioavailability) showed the greatest weight loss after this procedure because vitamin D supplementation exerted the strongest restorative effect on mineral homeostasis in this genotype group. In addition to its effect on calcium homeostasis, vitamin D regulates the transcription of numerous genes by activation of the nuclear vitamin D receptor [18]. Thus, it could be speculated that post-operative vitamin D supplementation regulated either the transcription of *FTO—*which encodes a N^6^-RNA demethylase [20]—or the transcription of genes that are functionally connected with *FTO* (e.g. *IRX3* or *RPGRIP1L*), or both, and that these effects of post-operative vitamin D supplementation on genetic regulation are strongest in vitamin D-deficient *FTO* A-allele carriers. However, the reader must keep in mind that these explanations are speculative and require further study.

Vitamin D can be synthesized by the human skin in response to sunlight exposure. Thus, vitamin D deficiency could also be a result of an inactive lifestyle, hallmarked by sedentarism and home-based activities (e.g., TV watching) [21–24]. Interestingly, a previous study involving patients who underwent bypass surgery has shown that patients significantly increase the time spent in leisure activities after the surgery [25]. With this in mind, it could be argued that RYGB surgery may have increased the time spent in leisure activities especially in those who were most sedentary before the surgery, i.e., obese patients who were vitamin D-deficient. Of note, physical activity has been shown to counteract the impact of *FTO* on body weight in humans [26]. In the same way, physical activity may boost the weight loss after RYGB surgery in AA carriers. However, bearing in mind that our study was of correlative nature, caution is needed before any firm conclusion can be drawn about the mechanism through which vitamin D may influence the impact of *FTO* on weight dynamics in humans.

As of yet, there is not much evidence for a beneficial effect of vitamin D supplementation on body weight regulation in humans. In a series of randomized controlled human studies on vitamin D supplementation, only one involving 36,282 post-menopausal women demonstrated that the supplementation of calcium plus vitamin D had a minimal but consistent favorable effect on weight (reviewed in [27]). However, these studies did not control for *FTO* [27], which may explain why the majority failed to show any beneficial effects of vitamin D supplementation on weight. Alternatively, the putative lack of efficacy of vitamin D supplementation in body weight reduction may also hint to vitamin D status as a simple marker of a sedentary lifestyle. This notion would also be in accordance with the previously found association of vitamin D deficiency and metabolic disease like type 2 diabetes [28, 29].

### Strengths and Limitations

Strengths of this study are that the study cohort was treated in a highly standardized fashion in a single center, thereby largely eliminating the biasing influence of distinct post-surgical care. Furthermore, since respective data were systematically collected, all analyses in our study could be adjusted for potential confounders such as age, sex, BMI before surgery, specific type of surgery, and season of vitamin measurement. However, also several limitations of our study need to be stated. Due to the 2-year follow-up time of our study, it remains unclear whether the observed interplay of *FTO* and vitamin D with weight loss persists over a longer follow-up period. Another limitation is that possible confounds by other factors, such as physical activity and the interplay of *FTO* with other genes [30, 31], which were not considered in the present analysis, cannot be excluded. Finally, generalization of our findings to other age groups (e.g., obese children undergoing bariatric surgery) or ethnic groups may not be appropriate.

## Conclusions

Our study suggests that the presurgery vitamin D status of patients undergoing RYGB surgery might be crucial for subsequent weight loss, especially in those who carry two copies of the *FTO* rs9939609 A-allele. Our findings could also offer a possible explanation as to why previous studies investigating the link between *FTO* and weight dynamics in humans have produced somewhat inconsistent results [6, 14, 15], as the vitamin D status of participants before starting the weight loss intervention may have not been taken into account in these studies. Supporting this view, in our cohort, nearly half of the obese patients exhibited a vitamin D deficiency before surgery, demonstrating that this micronutrient deficiency is abundant in obese people [32, 33].
